# Movable Layer Device for Rapid Detection of Influenza a H1N1 Virus Using Highly Bright Multi-Quantum Dot-Embedded Particles and Magnetic Beads

**DOI:** 10.3390/nano12020284

**Published:** 2022-01-17

**Authors:** Islam Seder, Ahla Jo, Bong-Hyun Jun, Sung-Jin Kim

**Affiliations:** 1 Department of Mechanical Engineering, Konkuk University, Seoul 05029, Korea; islam@konkuk.ac.kr; 2 Department of Bioscience and Biotechnology, Konkuk University, Seoul 05029, Korea; iamara0421@konkuk.ac.kr

**Keywords:** microfluidics, quantum dot, immunoassay, magnetic bead

## Abstract

Preventing the rapid spread of viral infectious diseases has become a major concern for global health. In this study, we present a microfluidic platform that performs an immunoassay of viral antigens in a simple, automated, yet highly sensitive manner. The device uses silica particles embedded with highly bright quantum dots (QD^2^) and performs the immunoassay with a vertically movable top layer and a rotating bottom layer. Through the motion of the layers and the surface tension in the liquids, reagents move from top chambers to bottom chambers and mix homogeneously. A tip in the top layer with a mobile permanent magnet moves the immune complexes comprising the magnetic beads, virus particles, and QD^2^ between the bottom chambers. In this way, our automated device achieves a highly sensitive magnetic bead-based sandwich immunoassay for the influenza A H1N1 virus within 32.5 min. The detection limit of our method is 5.1 × 10^−4^ hemagglutination units, which is 2 × 10^3^ times more sensitive than that of the conventional hemagglutination method and is comparable to PCR. Our device is useful for the rapid and sensitive detection of infectious diseases in point-of-care applications and resource-limited environments.

## 1. Introduction 

Virus detection on microfluidic platforms can be useful to prevent and control large outbreaks of infectious disease [1]. Nucleic acid testing and immunoassays are the main methods of virus detection [2,3] and have been successfully implemented on microfluidic platforms [4,5,6,7]. Nucleic acid testing that uses polymerase chain reaction has high detection sensitivity and specificity [8,9]. However, the requirements of temperature control and optical detection limit its use in resource-constrained environments. Among the immunoassays, one method allows the direct detection of viral proteins or antigens and is simple and fast [10,11]. This method does not involve time-consuming sample preparation steps and a complex apparatus and can obtain assay results within half an hour. However, it is less sensitive than nucleic acid testing [9]. To improve the sensitivity of direct virus detection, several detection methods have been developed such as plasmonic photothermal [12], electrochemical [13], and field-effect transistor-based biosensing [14]. Nonetheless, such detection methods lack robustness and platform reproducibility [15]. The fabrication and automation of microfluidics and the prevention of nonspecific binding of proteins in complex media also remain challenging in many cases [16]. Hence, rapid and accurate diagnosis requires the development of a highly sensitive, reproducible, and user-friendly method suitable for microfluidic immunological virus detection.

Quantum dots (QDs) are fluorescent semiconductor nanocrystals with unique optical properties including wide excitation bands and size-controlled emission in the visible spectrum. Compared with conventional organic dyes, QDs have high photostability for long-term analysis [17]. In our previous study, we developed a highly bright QD-embedded silica particle (QD^2^), which has 500 times stronger photoluminescence than individual QDs [18]. This QD^2^ was successfully applied to a magnetic bead (MB)-based sandwich immunoassay for influenza A H1N1 (FluA/H1N1) virus detection with higher sensitivity yet in a manual manner [19]. However, the manual process of adding, mixing, and washing an immune complex consisting of magnetic beads and QD^2^ is laborious, and the possibility of human error is high.

To apply the MB-based sandwich immunoassay in microfluidic devices and to make the assay process automated, numerous microfluidic components and peripheral setups are necessary. To date, several platforms have been studied [20,21,22,23,24]. Passive operation driven by capillarity [25] and vacuum [26] can simplify devices and their peripheral setups. However, such passive techniques lack customization for manipulating reagents and MBs [26,27]. To control them in a more sophisticated and automated manner, microfluidic devices typically require a complex arrangement and system integration for on-chip components such as active pumps [22], mixers [28], and valves [29]. However, such integration creates a bulky system and increases the cost of off-chip controllers. In a previous study [30], we presented a microfluidic device with open chambers in movable top and bottom layers. The device structure enabled us to control the mixing and pumping of reagents and MBs precisely without using on-chip pumps, mixers, and valves. However, it did not realize sample-to-answer detection for virus antigens. Moreover, a solution could be moved only partially from the top chamber to the bottom chamber with a detrimental loss of the solution.

**Scheme 1:**
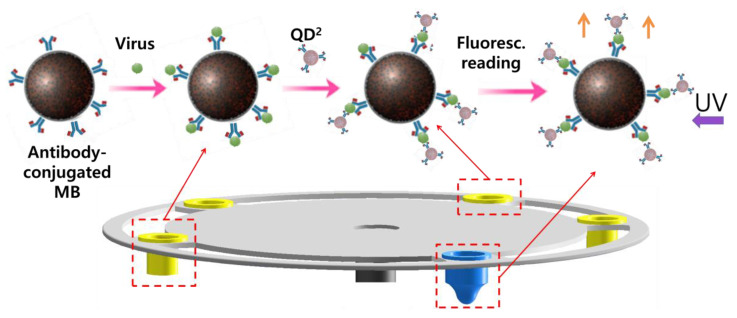
Schematic illustration of sandwich immunoassay-based virus detecting system applied on the movable chip.

**Figure 1:**
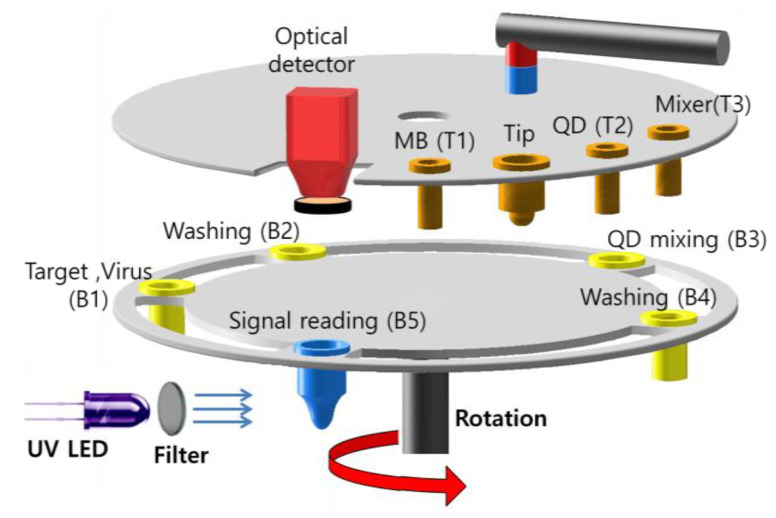
Schematic of the device with vertically movable top and rotating bottom layers. The top layer has three disposable chambers (T1–T3) and a disposable tip. The bottom layer has five disposable chambers (B1–B5). T1 and T2 contain antibody-conjugated magnetic bead (MB) and quantum dot-embedded silica particle (QD^2^) solutions, respectively. T3 is a hollow chamber. B1 contains a virus sample, and B2–B5 contain PBS buffer. An optical detector and an LED are integrated on the device for virus detection using quantum dot probes.

In this study, we present a microfluidic device that isolates viral antigen and detects the influenza A H1N1 virus by sandwich immunoassay, in a highly sensitive and automated manner. This device utilizes the high sensitivity of MB with the highly bright QD-embedded silica particle (Scheme 1) and the structural simplicity of the movable-layer device, and realizes the sample-to-answer system with the capability of on-chip detection (Figure 1). The vertical motion of the top layer allows its open chambers to contact the open chambers at the bottom layer, allowing complete reagent transport to the bottom chamber. The rotation of the bottom layer changes the location of the bottom chambers for the next interaction between the chambers in the top and bottom layers. A tip with a mobile permanent magnet on the top layer is used to collect, transfer, and release MBs. While carrying out the immunoassay of the tested virus, the device performed the collection of the virus, washing of the bead–virus–QD^2^ complex, and detection of the virus in an automated and effective manner.

## 2. Methods

### 2.1. Device Structure

For the device operation, disposable top (T1–T3) and bottom (B1–B4) chambers were placed on permanent top and bottom layers, respectively (Figure 1 and Figure S1). Figure 1 shows a schematic of the device operation and design. Chamber T1 has antibody-conjugated MBs, T2 has antibody-conjugated QD^2^s, and T3 is a stirrer for mixing. The disposable tip on the top layer is a hollow dome to load a permanent magnet, which moves vertically inside the tip to control magnetic strength. Bottom chamber B1 contains a viral sample, B2 has washing buffer 1, B3 has a buffer to incubate the complex of MB–virus–QD^2^, B4 has washing buffer 2, and B5 has a buffer for detection. Chambers T1–T3 are two-sided open cylinders, and chambers B1–B5 are topside open chambers. The position of each chamber is arranged carefully to avoid undesired contact during the layer motion. The vertical motion of the top layer and permanent magnet and the rotation of the bottom layer are independently controlled by three stepper motors. To excite and detect the fluorescence signal of QD^2^, an LED and a photomultiplier tube (PMT) are located at the side of B5 and at the top layer, respectively.

### 2.2. Device Fabrication and Peripheral Setups

Structural parts including the top and bottom layers, a magnetic arm, and a top supporting structure were fabricated with a filament (silver metallic PLA) by 3D-printer 1 (Ultimaker3, Ultimaker BV, Utrecht, The Netherlands). For the disposable chambers and the tip, we used various materials to print chamber A (ABS-like resin, Cubicon, Seongnam, Korea), chamber B (Acrylic yellow + resin, Cubicon), chamber C (Cubicon ABS, Esun, Shenzhen, China), and chamber D (MODELING TAN V2, Apply Labwork, Torrance, CA, USA). To manufacture the chambers, 3D-printer 2 (3DP-110DS, Cubicon) was used for chambers A and B, 3D-printer 3 (Single plus, Cubicon) for chamber C, and 3D-printer 4 (Form 3, Formlabs, Somerville, MA, USA) for chamber D. The 3D-printed chambers were exposed to UV light for 15 min and were left in an oven at 65 °C for 48 h. Chamber E was a transparent 200-μL commercial polypropylene tube (MicroAmp Fast Reaction Tube, Applied Biosystems, Waltham, MA, USA). To prevent the adsorption of virus particles, MB, and QD^2^ on the chamber surface, the disposable chambers were coated with a 5% bovine serum albumin (BSA) solution and were washed by a PBS buffer. We selected the 3D printing method for its ease of design and fabrication and its ability to iteratively modify and customize with device components during the research phase. However, for mass production of the chambers and commercialization of the chip, micro-milling and polymer injection molding can be used [31]. Because of the structural simplicity of our system, such mass production methods would be easily applied.

To control the vertical motion of the top layer and the permanent magnet [3 (φ) × 15 (L) mm^2^], two linear stepper motors (NEMA-23 and NEMA-17, respectively; HongYi Automation, Guangzhou, China) were used. To control the rotation of the bottom layer, a stepper motor (NEMA-17, Guangzhou Shenglong Motor, Guangzhou, China) was used. The layer motions were coded in open-source software (IDE, Arduino) and uploaded on a microcontroller (ATmega328, Arduino, Somerville, MA, USA) to control the three stepper motors automatically. For the excitation of QD^2^ fluorescence, a UV LED (VAOL-5EUV8T4, Visual Communications Company, Carlsbad, CA, USA) with a wavelength of 380 nm was positioned with an aspheric lens (83–677, Edmund Optics, Barrington, IL, USA) at the side of chamber B5 (Figure 1). The QD^2^s signals were obtained by PMT (H10722, Hamamatsu, Shizuoka, Japan). A filter with wavelength of 620 nm (FAS-Nano Amber Filter, NIPPON Genetics, Tokyo, Japan) was fixed on the top layer as a detector filter and its center wavelength was designated depending on the emission wavelength of QD^2^s fluorescent (625 nm). Details of the device components can be found in Table S1. The voltage output of the PMT was recorded and displayed on the Arduino microcontroller and software interface (Arduino IDE). Arduino divides the input voltage from the PMT into 1023 portion where 0 and 5 voltage input displays 0 and 1023 on software interface, respectively. The device was enclosed by a structure with a black color to prevent external constantly changing luminous intensity from interfering with the PMT reading. Sampling frequency of the PMT was 9.6 kHz. For each sample, the data obtained by the PMT were averaged and converted into one value. When we changed the sampling frequency from 9.6 to 6 kHz, the value of fluorescence intensity changed less than 2.8%. 

### 2.3. Preparation of Immunoassay Reagents

Virus samples comprising influenza A (FluA/H1N1 and FluA/H3N2: (California/07/2009/H1N1 and Texas/50/2012/H3N2) and influenza B (FluB/Yamagata: Massachusetts/02/2012 Yamagata lineage) were kindly provided by the Korea Institute of Radiological & Medical Sciences. The amount of virus was quantified using a hemagglutinin assay. We added the FluA/H1N1 virus in an erythrocyte solution with twofold dilution sequentially. The titer value was determined to be 64 hemagglutinating unit (HAU) (2^6^-fold dilution) by comparison with the negative control group without virus. In viral hemagglutination assay, one HAU is the amount of virus that will agglutinate 50% of the red blood cells in the standard agglutination assay (equal to approximately five–six logs of virus) [32]. The buffers used in chambers B2–B5 were PBS solutions. We estimated the analytical sensitivity for streptavidin and the virus particles using the limit of detection (LOD). For this task, blank determination method was used to obtain a nonzero standard deviation [33]. LOD evaluated the concentration that corresponded to the mean background intensity (I_B_) of the blank solution plus three times the standard deviation (SD): I_B_ + 3SD. The blank solution was PBS solution. We used CdSe@ZnS QD to make QD^2^ for high sensitivity immunoassay platform. MBs and QD^2^ were fabricated according to the protocol of our previous study [19]. More details for the fabrication process and the TEM imaging, UV spectrum, and SEM of silica nanoparticles and magnetic beads can be found in Figures S2–S4.

## 3. Results and Discussion

### 3.1. Assay Process

**Figure 2:**
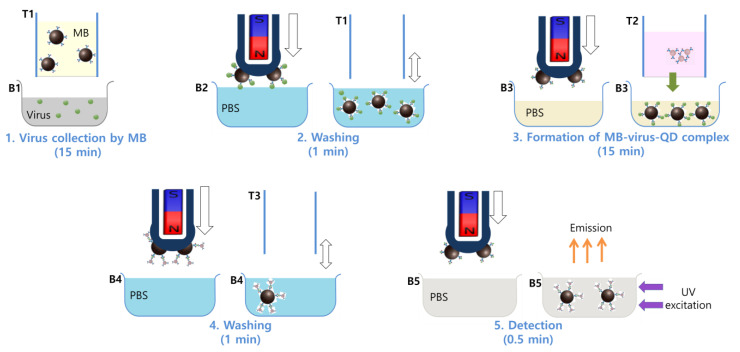
Schematic illustration of the sandwich immunoassay. The process consists of five steps. The reagent volumes are 70 μL each for the MB solution (T1), QD^2^ solution (T2), viral sample (B1), and PBS buffer (B3 and B5), and 210 μL for each washing buffer (B2 and B4).

After inserting the disposable chambers and the tip on the permanent layers, reagents were loaded in chambers T1 and T2 with volumes of 70 μL and in B1–B5 with volumes of 70, 210, 70, 210, and 70 μL, respectively. Next, the device automatically performed the assay process (Figure 2). In Step 1, to collect virus particles with MBs, chamber T1 moved down and touched B1. At this time, the MB solution was moved to the virus sample by surface tension (Figure S5). To enhance the virus collection with MBs by fluidic motion, T1 moved up and down repeatedly in B1 with a speed of 20 mm/s. In Step 2, the complexes of MB–virus were washed in B2. For this process, the tip with the permanent magnet collected the complexes and moved them to B2 by the vertical motion of the top layer and the rotation of the bottom layer. To release the complexes from the tip, the permanent magnet moved up, separated from the tip, and removed its magnetic force at the tip (Figure S6 and Movie S1). T1 was then brought back above B2 and moved up and down in B2 repeatedly to enhance the washing process of the complexes in B2. In Step 3, the complexes of MB–virus–QD^2^ were formed in B3. First, the tip transferred the complexes of MB–virus from B2 to B3. Next, the antibody-conjugated QD^2^s were moved from T2 to B3 by the contact between T2 and B3, and the complexes of MB–virus–QD^2^ were formed in B3. By its repeated vertical motion, T2 enhanced the formation of the complexes in B3. In Step 4, the complexes were washed in B4 to reduce the background signal. For this step, the tip moved the complexes from B3 to B4. Subsequently, instead of using the tip to enhance the washing process of the complex in B4, we additionally used T3, which is a hollow cylinder. This is because T3 showed better mixing efficiency than the tip (Figure S7 and Movies S2–S4). For the washing process, the bottom layer was rotated to align B4 and T3. T3 then vertically moved up and down and agitated the complexes in B4. In the last step, the complexes were moved to B5 by the tip and were detected. In Steps 1 and 3, each incubation time was 15 min, unless otherwise noted. For the experiment with streptavidin–biotin, the antibody and the virus were replaced by biotin and streptavidin, respectively.

Owing to the structural design of the separated chambers, the device executes multiple steps of reagent transporting and mixing with a minimum possibility for contamination among reagents. Motional precision and robustness of the top and bottom layers are ensured by stepper motors. Moreover, because of the simplicity of the device operation, the device has a low possibility of maintenance issues. 

### 3.2. Mechanism of Fluid Transport

The liquid is transferred from chamber T1 to B1 (Figure S5a). T1 is a top chamber with open top and bottom sides, and B1 is a bottom chamber with an open top side. After contact, the top chamber moves up and drains the liquid to the bottom chamber by surface tension. To understand the process, we fixed the bottom chamber diameter to 6 mm and varied the top chamber diameter from 2 to 5 mm. We define the diameter ratio as *D*_T_/(*D*_B_ − *D*_T_), where *D*_T_ and *D*_B_ are the diameters of the top and bottom chambers, respectively. After the contact of the two chambers, liquid remained in the top chamber when *D*_T_/(*D*_B_ − *D*_T_) < 1.9. However, for *D*_T_/(*D*_B_ − *D*_T_) ≥ 1.9, the liquid was completely transferred to the bottom chamber (Figure S5b). This result is affected by surface tension of the liquid under the influence of gravity. To explain the result qualitatively, we developed a model that shows the ratio of surface tension forces (*F*_L_/*F*_U_) at the moment when the top chamber rises up after the contact, where *F*_L_ (*F*_U_) is the surface-tension force that attracts down (up) the liquid meniscus at the bottom (top) side of the top chamber (Figure S5c). Details of the model are shown in supporting information. The ratio of the surface-tension forces from the developed model is:

(1) $$\left|\frac{F_{L}}{F_{U}}\right|\;\sim \;\left(\frac{D_{T}}{D_{B}-D_{T}}\right).$$

In addition, for the influence of gravity on the fluid drainage, we studied the Bond number *Bo* = *ρgD*_T_^2^/*σ*, which is the force ratio of gravity to surface tension. Here, *ρ* and *σ* are the density and surface tension of the solution, respectively, and *g* is gravity. With increasing *D*_T_, *Bo* increased from 0.1 to 2.2. Thus, as *D*_T_ is increased, the gravity further promotes the fluid transfer.

### 3.3. Enhancement of the Detection Sensitivity

**Figure 3:**
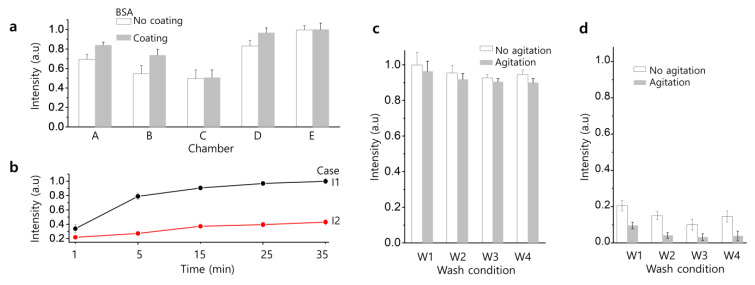
Characterization of on-chip sandwich immunoassay. Error bars are S.D. for *n* = 3. (**a**) Influence of the chamber material on virus detection. Chambers A–D are 3D-printed with different materials, and chamber E is a commercial polypropylene tube. (**b**) Effect of the incubation time and agitation on the binding between the MBs and the virus particles in Step 1 of Figure 2. Cases I1 and I2 represent continuous agitation and agitation for the first 6 s, respectively. (**c**) Influence of the washing procedure in Step 4 to remove the background signal noise for virus detection. The fluorescence intensities are shown with the virus in (**c**) and without the virus in (**d**). Under conditions W1, W2, and W3, one, two, and three chambers of B4 with a 70 μL volume each are used, respectively, and under condition W4, one chamber of B4 with a 210 μL volume is used.

We studied the influence of the chamber material, incubation time, and washing procedure on virus detection to enhance the detection sensitivity. For all the cases, we used FluA/H1N1 virus suspension with a concentration of 3.2 × 10^−1^ HAU. First, we examined chambers A–E manufactured using various materials to prevent the adsorption of the MBs, QD^2^, and virus particles on the chamber surfaces. Once we selected a chamber with a specific material, it was used in Steps 1–4 (Figure 2). In Step 5, chamber E was used for the detection of the complex of MB–virus–QD^2^. Figure 3a shows the fluorescence intensity of the complex in Step 5. The BSA coating in chambers A and B increased the fluorescent intensities by 17.4% and 25.4%, respectively. This indicates that the BSA coating prevented the adsorption of the MBs, QD^2^, and virus particles on the chamber surfaces in Steps 1–4. However, regardless of the BSA coating, the fluorescent intensities in chambers A–C were lower than those in chambers D and E. As chamber D with the BSA coating produced a similar result to that of chamber E—which was the commercial tube—we used the BSA-coated chamber D in Steps 1–4. 

Next, to enhance the conjugation between the MBs and the virus particles in Step 1, we varied the incubation time and the agitation condition. Two cases were considered for the agitation: continuous agitation by the vertical motion of chamber T1 at a speed of 20 mm/s (Case I1) and agitation only for approximately the first 6 s to ensure an initial homogeneous suspension (Case I2). Figure 3b shows that, for both cases, the fluorescence intensity increased in Step 5 as the incubation time in Step 1 was increased. However, Case I1 significantly enhanced the intensity, as compared with Case I2. The normalized intensity rapidly increased to 0.9 for 15 min and then gradually reached 1 for the next 20 min. When we performed the incubation manually and increased the incubation time from 30 min to 3 h, the fluorescence intensity increased only by 7.3% (Figure S8). Thus, for effective agitation, considering the intensity enhancement and the short assay time, we chose a 15-min agitation process in Step 1 under Case I1.

We also studied the effect of washing in Step 4 on virus detection. In Step 4, we varied the number of washing procedures and the volume of the washing reagent. For conditions W1, W2, and W3, one, two, and three chambers of B4 with a 70-μL volume in each were used, respectively, and for condition W4, one chamber of B4 with a 210-μL volume was used. Under these conditions, we compared the influence of the agitation by the vertical motion of T3 at a 20 mm/s speed. If the agitation was not performed, after the tip released the complex of MB–virus–QD^2^ to chamber B4, the tip agitated chamber B4 only for 6 s to homogenize the suspension. When we used the viral suspension, the agitation slightly decreased the fluorescence intensity in Step 5 by 4% regardless of the washing conditions (Figure 3c). However, for the negative control group (without the virus particles), the agitation significantly reduced the intensity under different washing conditions (Figure 3d). Specifically, for washing conditions W1, W2, and W3, the intensity decreased to 0.10, 0.04, and 0.03 (reduction of 54%, 72%, and 70%), respectively. This result indicates that the agitation can effectively wash unbound QD^2^ on the complex in Step 4, thereby significantly decreasing the background signal in Step 5. However, increasing the number of washing steps requires additional chambers on the device, complicating the device structure. Under condition W4, after washing once with single B4 in three times its volume, the fluorescence intensity was similar to that under conditions W2 and W3. Thus, we applied condition W4 for effective washing. Through the process optimization, we could significantly reduce the assay time from 1 h to 32.5 min compared with our previous study [19].

### 3.4. Performance of the Immunoassay on the Movable Device 

To investigate the sensitivity of the QD^2^-based immunoassay using the movable-layer device, we used the interaction of streptavidin and biotin as a model system because of their high affinity and specificity. We applied a wide range of streptavidin concentrations and used biotinylated MB and biotinylated QD^2^ solutions. The steps for the test are specified in Figure 2. The device can cover the streptavidin concentration in the range of 1 µM–1 zM (Figure S9).

**Figure 4:**
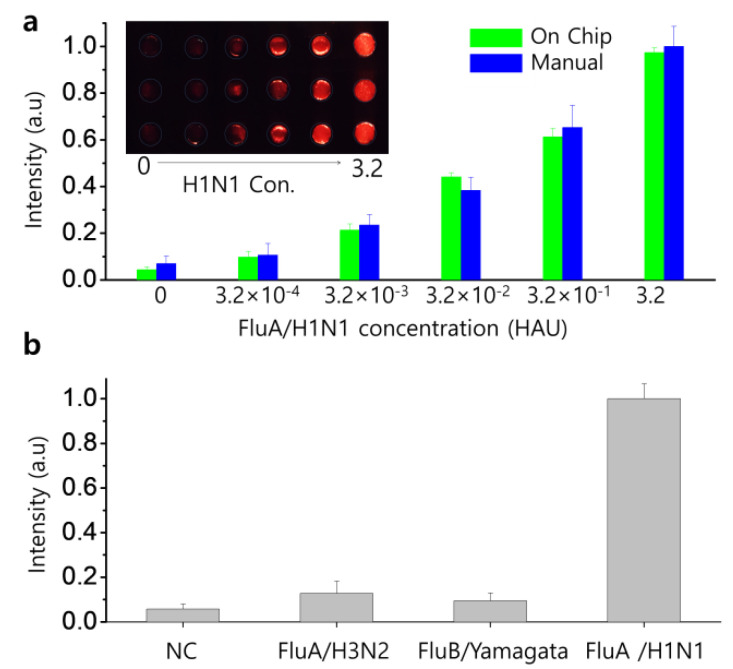
On-chip virus detection using the QD^2^-based sandwich immunoassay. Error bars are S.D. for *n* = 3. (**a**) Fluorescence-based detection of the influenza A H1N1 virus (FluA/H1N1). Optical intensity quantified by an optical detector (PMT) for the automated device and manual operation. The inset shows a photo of an array with the immune complex under UV light. (**b**) Selectivity of the on-chip immunoassay. The fluorescence intensities are compared between the target virus FluA/H1N1 and nontarget viruses including influenza A H3N2 (FluA/H3N2) and influenza B Yamagata lineage (FluB/Yamagata).

The sensitivity of our device-based immunoassay was then assessed for the detection of the FluA/H1N1 virus. Figure 4a quantifies the fluorescence intensities obtained using our automated device and the manual method. The fluorescence intensities of the two methods agreed well over a wide range of 3.2 × 10^−4^–3.2 HAU. Negative control experiments were performed without the virus. The LOD of the device was 5.1 × 10^−4^ HAU. Compared with the viral hemagglutination assay with its LOD of 1 HAU [32], our method provides 2 × 10^3^ times higher sensitivity. The LOD obtained by our device is similar to the LOD obtained with the aptamer assay [34]. Because patient samples (blood, salvia, and nasal swab) express the viral load in a wide dynamic range, depending on the patient’s health condition and hospitalization onset [35,36,37], direct comparison with the patient is impractical. The sensitivity of the immunoassays for the presence of antibodies in human samples may also depend on the viral titer and time of sample collected after viral infection; both factors impact circulating antibody concentration [10]. Although the LOD of our device is 16 times less sensitive than that of the real-time PCR [19], the turnaround time of our assay is shorter (33 min), and the assay process is simpler without complex sample purification and thermal cycling. We compared the sensitivity of our method with other studies in Table S2. To visualize the fluorescence variation, we collected the complexes of MB–virus–QD^2^ and moved them to an array with a UV source. This allowed us to simultaneously visualize complexes at widely different concentrations. The photo of the array taken by a smartphone shows that even observations with a naked eye can clearly distinguish the low concentration of FluA/H1N1 virus down to 3.2 × 10^−3^ HAU (Inset of Figure 4a). This suggests that our method can provide assay results that are observable with the naked eye, without the use of expensive optical detectors in resource-limited settings. The interference study using various real samples with UV absorption and fluorescence is necessary for future works for practical clinical applications. This is because biomatters in blood, serum, cells, marine water, and waste waters display strong background UV absorption and fluorescence that may weaken the fluorescence signal for target detection.

### 3.5. Specificity Tests of the Integrated Microfluidic System

To evaluate the specificity of our sandwich immunoassay device, we performed assays with the same steps shown in Figure 2 with different viruses (FluA/H3N2 and FluB/Yamagata). Figure 4b shows that the fluorescence signals from the FluA/H1N1 virus is at least eight times higher than the signals from FluA/H3N2 and FluB/Yamagata. Here, we used the same concentration of 3.2 × 10^−1^ HAU. This result indicates that our method can reliably differentiate the target virus from others.

## 4. Conclusions

Sandwich immunoassay using highly bright multi-QD embedded particles (QD^2^) and MBs was successfully applied on a device with movable layers. The movable-layer device enabled multiple fluid manipulation steps including fluidic transport, mixing, and MB manipulation without any on-chip valves and pumps in a simple and precise manner. With the use of QD^2^, the automated device had a high level of sensitivity and specificity for influenza A H1N1 virus detection within 33 min. The proposed device, which provides a high level of sensitivity and specificity for influenza A H1N1 virus detection in a fully automated manner, could help in the implementation of point-of-care testing. Currently, users need to apply reagents to the device. However, with the improvement of the device design, reagents could be preloaded and stored in the device to facilitate its use. With the improved level of sensitivity and turnaround time of the immunoassay, the proposed fluid manipulation technique can be applied to a wide range of immunoassay applications.

## Data Availability

The data presented in this study are available on request from the corresponding author.

## Supplementary Materials

The following supporting information can be downloaded at: https://www.mdpi.com/article/10.3390/nano12020284/s1, Figure S1: Permanent layer and disposable chamber. (a) 3D-printed permanent layer. Disposable chambers can be placed in the layer. The inset shows a magnified image of the seat for a disposa-ble chamber. (b) 3D-printed disposable chamber that is inserted in the seat. (c) Chamber B5 that is used for optical detection. (d) Real image of the device; Figure S2: TEM imaging and UV spectrum of silica nanoparticles; Figure S3: SEM images of (a,b) magnetic beads and (c,d) silica coated magnetic beads. (a,c) are high-magnitude SEM images and (b,d) are low-magnitude SEM images; Figure S4: TEM images of QD2 (left) and UV-Vis spectroscopy of QD2 (right); Figure S5: Fluidic transport by surface tension. (a) Liquid transfer from the top to bottom cham-bers by the contact between the solutions in T1 (red color) and B1 (blue color). (b) Real images of fluid transfer from top to bottom upon varying the top chamber diameter. (c) Model of the mechanism of liquid transport. (i) Photo showing the meniscus when the liquids in the top and bottom chambers come into contact. (ii) Surface tension and gravitational forces; Figure S6: Manipulation of magnetic beads. A permanent magnet (white dotted line), located in-side the tip, moves together with the tip for 0–21 s. Next, the magnet moves up from the tip to remove the magnetic force; Figure S7: Mixing efficiency. (a) Photographs that show the mixing process. (b) Mixing efficiency in the bottom chamber. As the number of contacts between the bottom chamber and T3/tip is in-creased, the mixing efficiency increases. (c) Pressure profiles of the bottom chamber during one cycle of agitation; Figure S8: Change in the fluorescent intensity in the detection by off-chip incubation. The incubation time corresponds to the times in Steps 1 and 3 in each; Figure S9: Fluorescence intensity for biotin–streptavidin interaction. The QD2-based immunoassay was used as a model system to investigate the sensitivity. The red point shows the negative control (without streptavidin). The streptavidin LOD was found to be 0.39 aM. This result shows that the QD2 and MB-based sandwich immunoassay with our device enables highly sensitive detection; Table S1: Details of device component and its circuit; Table S2: Immunoassays using optical nanoprobes for virus detection; Movie S1: Bead collection; Movie S2: Bead mixing; Movie S3: Reagent transport; Movie S4: Reagent mixing.
