# Supplementary material for: Movable Layer Device for Rapid Detection of Influenza a H1N1 Virus Using Highly Bright Multi-Quantum Dot-Embedded Particles and Magnetic Beads
Source: Nanomaterials (Basel). 2022 Jan 17;12(2):284. doi: 10.3390/nano12020284 (PMC8778663; doi:10.3390/nano12020284)
Supplement: Supplementary file 1 [file nanomaterials-12-00284-s001.zip › nanomaterials-1522795-suppl.pdf]

nanomaterials

MDPI

Supplementary Information

Movable Layer Device for Rapid Detection of Influenza a H1N1 Virus Using Highly Bright Multi-Quantum Dot-Embedded Particles and Magnetic Beads

Islam Seder [1], Ahla Jo [2], Bong-Hyun Jun [2,*] and Sung-Jin Kim [1,*]

[1] Department of Mechanical Engineering, Konkuk University, Seoul 05029, Korea; islam@konkuk.ac.kr
[2] Department of Bioscience and Biotechnology, Konkuk University, Seoul 05029, Korea; iama-ra0421@konkuk.ac.kr
* Correspondence: bjun@konkuk.ac.kr (B.-H.J.); yahokim@konkuk.ac.kr (S.-J.K.) Tel.: +82-2-450-0521 (B.-H.J.); +82-2-450-0517 (S.-J.K.); Fax: +82-2-447-5886 (S.-J.K.)

Bottom Layer with a Disposable Chamber

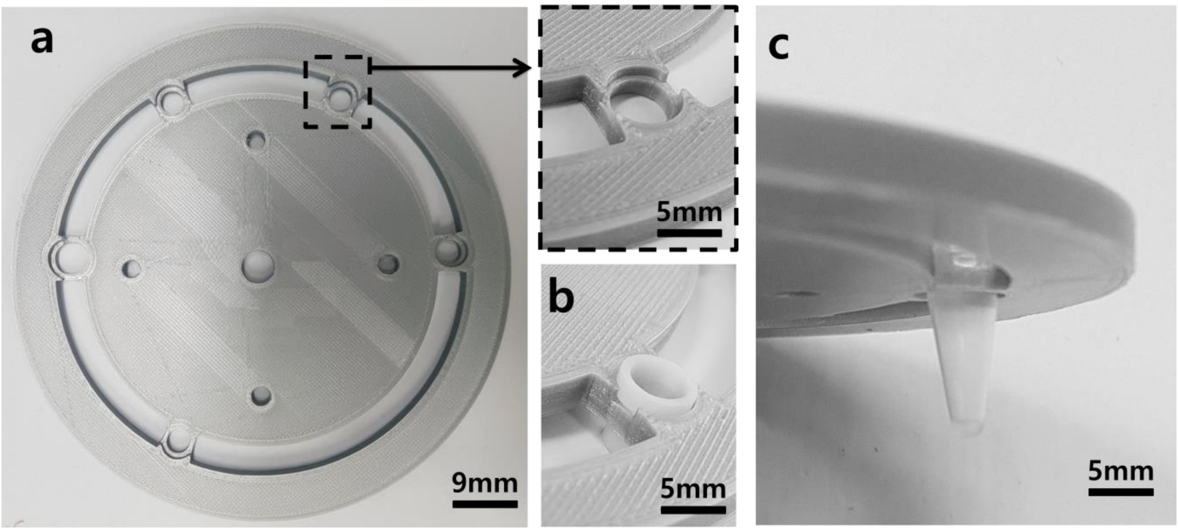

d

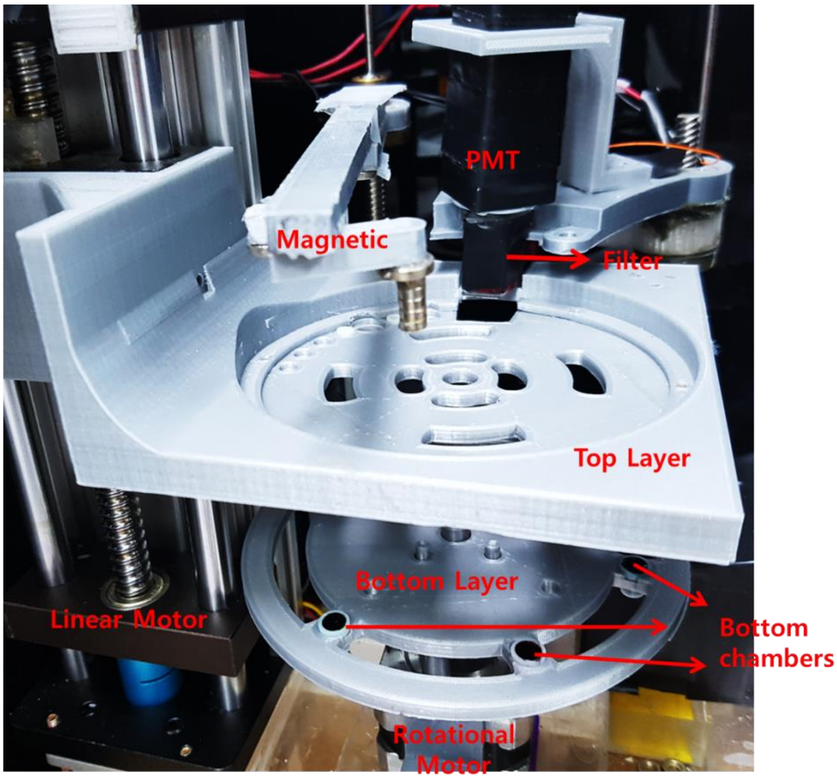

Figure S1. Permanent layer and disposable chamber. (**a**) 3D-printed permanent layer. Disposable chambers can be placed in the layer. The inset shows a magnified image of the seat for a disposable chamber. (**b**) 3D-printed disposable chamber that is inserted in the seat. (**c**) Chamber B5 that is used for optical detection. (**d**) Real image of the device.

Fabrication of QD-Embedded Silica Nanoparticles and Silica-Coated M-Beads

All reagents were used as received from the suppliers without further purification. Tetraethylorthosilicate (TEOS), 3-mercaptopropyltrimethoxysilane (MPTS), (3-aminopropyl)triethoxysilane (APTS), succinic anhydride, N,N-Diisopropylethylamine (DIEA), N-(3-Dimethylaminopropyl)-N′-ethylcarbodiimide (EDC) hydrochloride, N-Hydroxysulfosuccinimide (Sulfo-NHS) sodium salt, 2-(N-Morpholino)ethanesulfonic acid hydrate (MES), bovine serum albumin (BSA), phosphate buffered saline (PBS, pH 7.4), TWEEN® 20, and ethanolamine were purchased from Sigma Aldrich (New York, NY, USA). Phosphate buffered saline (PBS, pH 7.4) was obtained from Invitrogen (Carlsbad, CA, USA). CdSe@ZnS QDs were purchased from Zeus (Osan, SouthKorea). Ethanol (EtOH, 98%) and absolute ethanol (abs EtOH, 99.9%) were purchased from Daejung (Si-heung, Korea). Virus suspensions (influenza A virus/California/07/2009/H1N1, influenza A virus/Texas/50/2012/H3N2and influenza B/Massachusetts/02/2012 Yamagata lineage) cultured in allantoic fluid of embryonated eggs were provided by the Korea Institute of Radiological & Medical Sciences (Seoul, Korea).

MBs and QD^2 were fabricated according to our previous study [ref 19 in the main text]. To fabricate QD^2, aqueous ammonium hydroxide (NH_4OH) (28%, 3 mL) was added to EtOH (99.9%, 40 mL) containing tetraethyl orthosilicate (TEOS, 1.6 mL) with vigorous magnetic stirring for 1 day at room temperature. After washing with ethanol, 300 mg of the prepared silica particles (diameter: 120 nm) was dispersed in 12 mL of EtOH. Subsequently, the silica particles were modified by thiol with 600 µL of 3-mercaptopropyltrimethoxysilane (MPTS) and 120 µL of NH_4OH. Next, CdSe@ZnS QDs were immobilized on thiolated silica particles (10 mg) in EtOH with QDs (70 µg) in a dichloromethane solution (4 mL). Furthermore, MPTS (50 µL) and NH_4OH (50 µL) were added to the mixture and rotated for at least 30 min. The colloids were washed five times and dispersed in EtOH. Subsequently, QD-embedded silica particles (10 mg) were dispersed in EtOH (5 mL) and rotated for at least 12 h after adding MPTS (50 µL) and NH_4OH (50 µL). Finally, the colloids were dispersed in EtOH (5 mL). In our experiment, the size of the silica nanoparticles was approximately 120 nm and the silica nanoparticles were homogeneous and well-dispersed without aggregation. When the absorbance wavelength of the silica nanoparticles was measured in the range from 300 nm to 1100 nm, the absorbance decreased toward a long wavelength (Figure S2).

To fabricate silica-coated MBs, monodisperse porous polystyrene-divinylbenzene (PS-DVB) beads were prepared by the seeded emulsion polymerization method. To fabricate monodisperse macroporous PS-DVB, first, we prepared monodisperse PS seeds using the dispersion polymerization method. A total of 150 mg of 2,2′-azobis-isobutyronitrile was dissolved in 15 mL of inhibitor-removed styrene and was then added to the as-prepared dispersion medium, which was ethanol/2-methoxyethanol (3:2) containing polyvinylpyrrolidone-40 (1 g) as a steric stabilizer (90 mL). The mixture was sonicated for 10 min. Next, dispersion polymerization was performed in a cylindrical reaction chamber with shaking (120 cpm) at 70 °C overnight. The precipitates were washed intensively with distilled water and EtOH. The PS seeds were vacuum-dried overnight affording PS seeds (*ca* 4 µm). The PS seeds (700 mg) dispersed in dibutyl phthalate (DBP, 0.7 mL)-emulsified aqueous medium (100 mL) containing 0.25% sodium dodecyl sulfate (SDS) swelled with DBP for 20 h at room temperature. An aqueous medium (100 mL) containing a 0.25% SDS-emulsified mixture of styrene (4.6 mL) and DVB (2.3 mL) with dissolved dibenzoyl peroxide (BPO, 240 mg) was prepared by using a homogenizer for 1 min. The emulsified monomer solution was added into the DBP-swollen PS seed dispersion medium, and the swelling monomer was continuously stirred for 20 h. The DBP-swollen PS seed dispersion medium with the emulsified monomer solution was stirred for 20 h. Next, an aqueous solution of 10% PVA in deionized water (10 mL) was added. The medium was purged with nitrogen stream for 30 min. To obtain monodisperse PS-DVB beads, the seeded polymerization mixture was stirred at 70 °C for 20 h. Subsequently, the polymer beads were intensively washed with warm deionized water (50 °C),

EtOH, and THF to remove DBP and linear polymers. Finally, the macroporous PS-DVB beads (*ca* 8 μm) were dried in vacuum at 30 °C for 24 h. To sulfonate the PS-DVB beads, monodisperse PS-DVB beads (1 g) were added to acetic acid (5 mL) in an ice bath. The mixture was stirred between 30 min and 2 h after sulfuric acid (50 mL) was slowly added to the beads and the temperature was increased up to 90 °C. The dispersion reaction was quenched the into ice water (400 mL), and the sulfonated PS-DVB beads were collected by washing with deionized water and EtOH. Subsequently, the sulfonated microspheres were dried under vacuum (1.1 g). The sulfonated macroporous PS-DVB beads (500 mg) were dispersed in deionized water (10 mL). Next, a freshly prepared mixture of $FeCl_3{\cdot}6H_2O$ (618 mg, 2.26 mmol) and $FeCl_2{\cdot}4H_2O$ (257 mg, 1.28 mmol) was added to the dispersion solution for adsorption. After 2 h, the bead suspension was added with continuous stirring 28% NH_4OH (50 mL) for 40 min. The magnetized microspheres were washed with 25% trifluoroacetic acid. After that, they were extensively washed with deionized water and EtOH. Finally, the magnetized microspheres were dried under vacuum (magnetized microspheres). A total of 100 mg of the magnetic sulfonated PS-DVB beads, by adding an 3-aminopropyl tri-ethoxysilane (APTES) solution (1%, 100 mL), was shaken for 10 min and for 20 min after adding NH_4OH (28%, 2 mL). Thereafter, TEOS (2 mL) was added in the dispersion mixture and shaken for 12 h. The resulting beads (silica-coated MBs) were collected by the magnet and washed with EtOH. The size of bead was 8 μm and the bead exhibited superparamagnetic properties (Figure S3).

For the amination of the QD^2 and silica-coated MB surfaces, each QD^2 (1 mg) and MB solution (1 mg) were added in a 5% APTES solution and 1% NH_4OH. Subsequently, the colloids were dispersed in PBS (1 mL). Next, we biotinylated the aminated QD^2 and MB surfaces and added activated biotin (20 mg) to the aminated QD^2 (100 μg) and MBs (100 μg). Finally, to conjugate the antibody to the QD^2 and MB surfaces, we first modified the surface of the QD^2 by carboxyl groups. Next, amine-modified QD^2 (1 mg) were dispersed in *N*-methyl-2-pyrrolidone (NMP, 500 μL), followed by the addition of succininc anhydride (SA, 1.75 mg) and *N,N*-diisopropylethylamine (DIEA, 3.05 μL) to the mixture. The colloids were dispersed in *N,N*-dimethylformamide (DMF, 200 μL). The MB surface was modified by carboxyl groups. Next, amine-modified MBs (1 mg) were added to a mixture of a 5% APTES solution and 1% NH_4OH and then re-dispersed in NMP (500 μL). The colloid suspension was added to SA (1.75 mg) and DIEA (3.05 μL) and re-dispersed in DMF (200 μL). Next, the antibody (10 pmol) was conjugated to the surface of carboxylate QD^2 and MBs under the catalysis of 1-3-dimethylaminopropyl-3-ethylcarbodiimide hydrochloride (EDC)/*N*-hydroxysuccinimide (NHS).

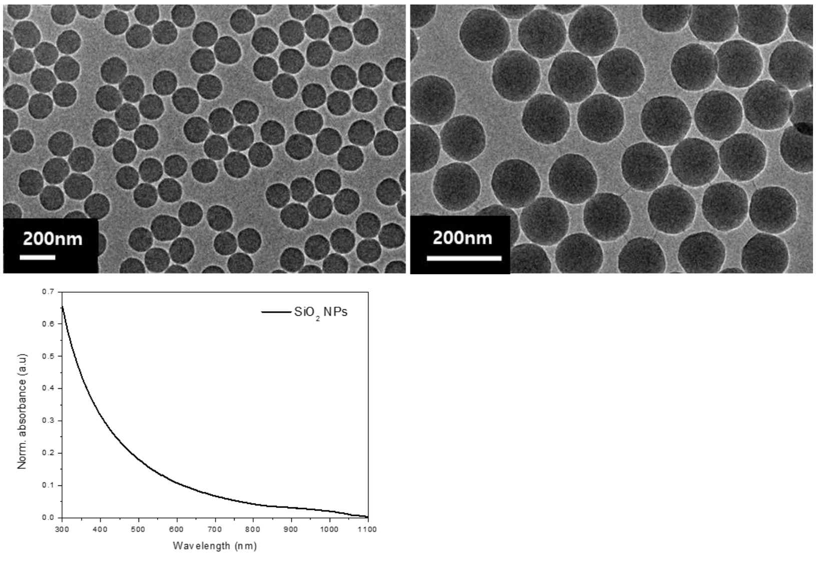

Figure S2. TEM imaging and UV spectrum of silica nanoparticles.

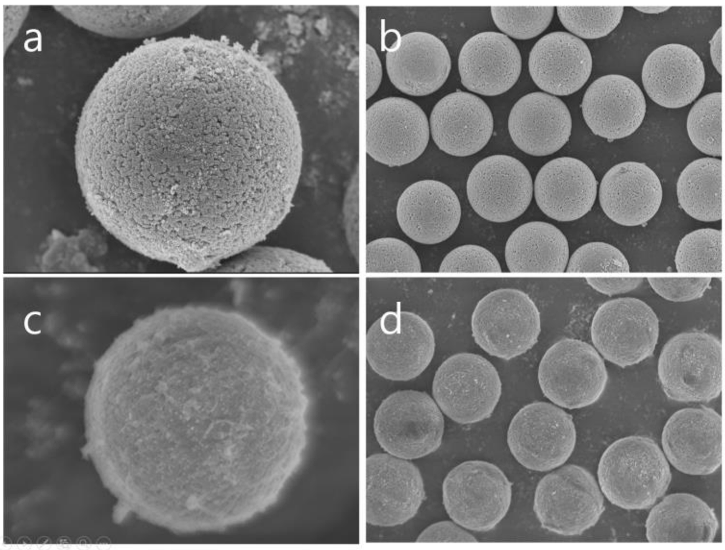

Figure S3. SEM images of (**a**,**b**) magnetic beads and (**c**,**d**) silica coated magnetic beads. (**a**,**c**) are high-magnitude SEM images and (**b**,**d**) are low-magnitude SEM images.

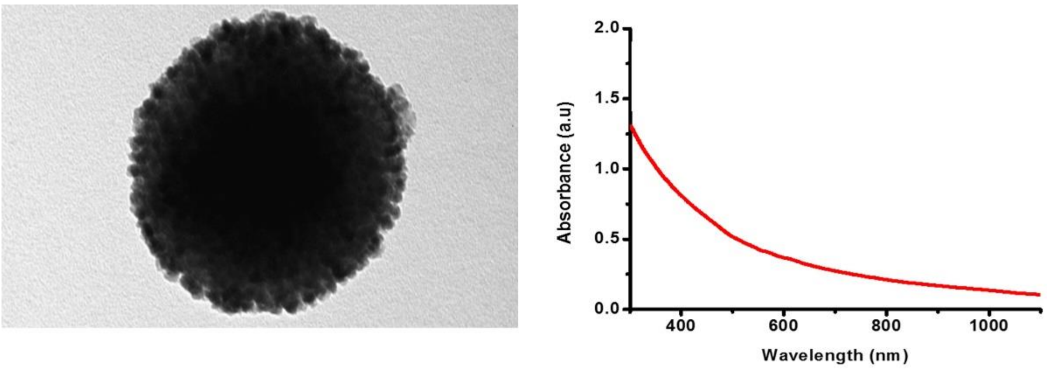

Figure S4. TEM images of QD^2 (**left**) and UV-Vis spectroscopy of QD^2 (**right**).

Mechanism of Fluid Transport by Surface Tension

To explain the mechanism of fluid qualitatively, we developed a model that shows the ratio of surface tension forces (F_L/F_U) at the moment when the top chamber rises up after the contact. Here, F_L (F_U) is the surface-tension force that attracts down (up) the liquid meniscus at the bottom (top) side of the top chamber (Figure S5c). When F_L/F_U is >> 1, the liquid in the top chamber is totally transferred to the bottom chamber. F_L and F_U are primarily influenced by the size and shape of the chambers and are proportional to $1/(D_B–D_T)$ and $1/D_T$, respectively. This results in the relation of $F_L/F_U \sim D_T/(D_B - D_T)$, which qualitatively agrees well with the experimental results.

The detailed equations are as follows. F_U is obtained from part A of Figure S4c:

$$F_{\mathrm{U}} \sim \frac{\sigma}{D_{\mathrm{T}}}\left(\frac{\pi}{4}D_{\mathrm{T}}{}^{2}\right). \tag{S1}$$

F_L can be calculated using part B of Figure S5b:

$$F_L \sim \sigma\left(\frac{-1}{r_1}+\frac{1}{r_2}\right)\left(\frac{\pi}{4}D_{\mathrm{T}}{}^{2}\right), \tag{S2}$$

where r_1 and r_2 are the principal radii of the meniscus. Note that F_L is less than zero because it pulls down the liquid of part A. From part B of Figure S5b, r_1 and r_2 can be approximated by

$$r_1 \sim D_{\mathrm{B}} - D_{\mathrm{T}} \ \text{ and } \ r_2 \sim D_{\mathrm{T}}. \tag{S3}$$

Using the result of $F_L < 0$, we can consider $\left|\frac{1}{-r_1}\right| > \left|\frac{1}{r_2}\right|$. Thus, equation S2 can be approximated by

$$F_{\mathrm{L}} \sim \sigma\left(\frac{-1}{D_{\mathrm{B}}-D_{\mathrm{T}}}\right)\left(\frac{\pi}{4}D_{\mathrm{T}}{}^{2}\right). \tag{S4}$$

From equations S1 and S4, the ratio of the surface-tension forces is

$$\left|\frac{F_{\mathrm{L}}}{F_{\mathrm{U}}}\right| \sim \left(\frac{D_{\mathrm{T}}}{D_{\mathrm{B}}-D_{\mathrm{T}}}\right). \tag{S5}$$

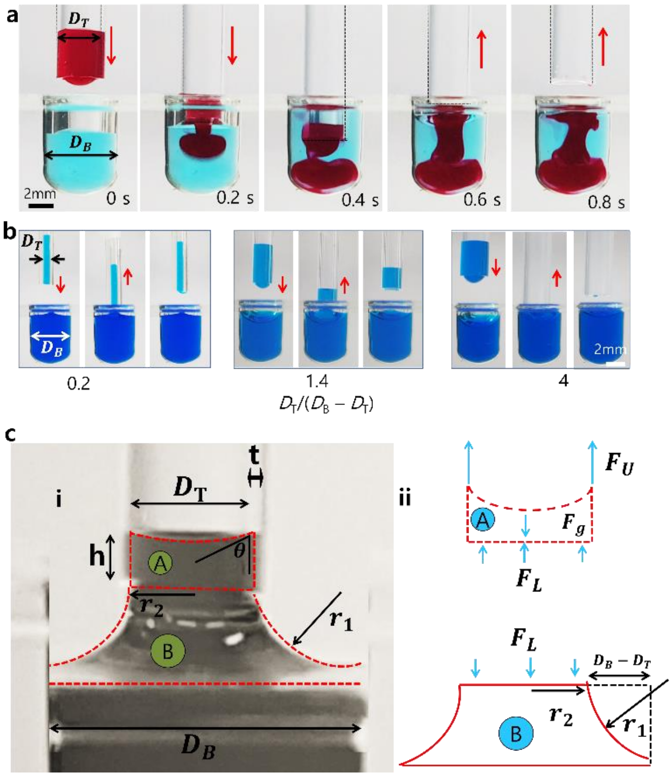

Figure S5. Fluidic transport by surface tension. (**a**) Liquid transfer from the top to bottom chambers by the contact between the solutions in T1 (red color) and B1 (blue color). (**b**) Real images of fluid transfer from top to bottom upon varying the top chamber diameter. (**c**) Model of the mechanism of liquid transport. (i) Photo showing the meniscus when the liquids in the top and bottom chambers come into contact. (ii) Surface tension and gravitational forces.

Collection and Transfer of Magnetic Beads

Biotinylated magnetic beads (MBs) were manipulated by using a vertically movable permanent magnet inside the tip. MBs were collected, transferred, released, and mixed in the operational chamber (Figure S6). To gather the complex, at time $t = 0$ s, the tip moved downward and contacted the liquid in the bottom chamber containing MBs. Next, at $t = 1$ s, a permanent magnet rod approached the tip to apply a magnetic force on the MBs and collect them. The collection took less than 20 s. For $t = 20–30$ s, the tip transported the MBs to a new bottom chamber and dropped them inside the chamber: the tip and the magnet moved upward and then downward together, while the bottom layer rotated to align the tip and the chamber. Later, the tip contacted the liquid in the chamber, and then the magnet rod separately moved upwards to remove the magnetic force at the tip, releasing the MBs in the chamber.

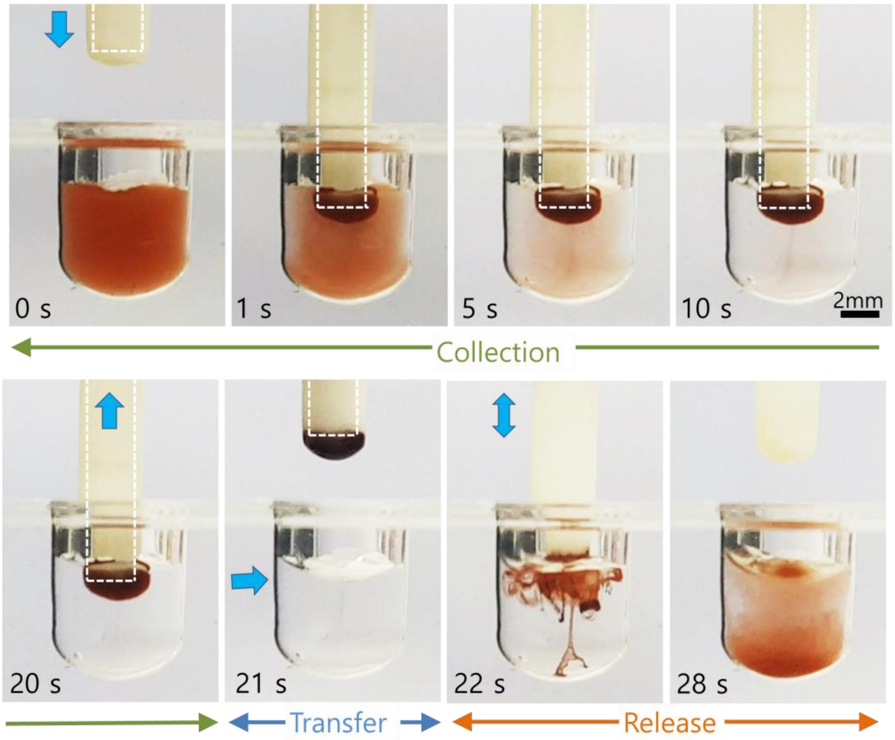

Figure S6. Manipulation of magnetic beads. A permanent magnet (white dotted line), located inside the tip, moves together with the tip for 0–21 s. Next, the magnet moves up from the tip to remove the magnetic force.

Characterization of the Mixing Efficiency

We compared the mixing efficiency between the tip and chamber T3. After the complexes of MB–virus–QD[2] were released to B4, the tip or T3 vertically moved up and down with a 20 mm/s speed to agitate the complex in B4. Figure S7a clearly shows that mixing with T3 is more effective than that with the tip. After 12 contacts, the mixing efficiency by the tip was only 67 %, whereas that by T3 was 98% (Figure S7b). To explain this result, we measured the pressure of B4 for the one cycle of the insertion and withdrawal of the tip and T3 at B4 (Figure S7c). The tip and T3 were separated from B4 in state A, moved down to B4 in state B, maintained their position inside B4 in state C, moved up from B4 in state D, and were separated again in state E. The pressure amplitude was 2.4 times higher by T3 than by the tip in states B and D (75.8 vs 31.3 Pa). This led to higher mixing efficiency by T3.

To measure the mixing efficiency, we used an MB solution with a 50 μL volume for the tip and T3 and a clear solution (100 μL, PBS) for B4. A stereo microscope (u-SZ40, Olympus) and a camera (u-Nova20C, Novitec) were used to take images at a rate of 50 fps. The captured images were analyzed using a software (Octave), and the mixing efficiency (η) was calculated:

$$\eta = \frac{\frac{1}{N}\sum_{i=1}^{N}(I_{\mathrm{i}}-I_{0\mathrm{i}})}{\frac{1}{N}\sum_{i=1}^{N}(I_{\mathrm{r}}-I_{0\mathrm{i}})} \times 100\%$$

where I indicates the normalized gray scale value, I_i is the value of each pixel at the ith pixel of the captured image, I_r is the normalized value of the reference image in the fully mixed state, N is the number of pixels, and I_{0i} is the normalized value at the *i*th pixel in the initial state, i.e., without mixing. Thus, η = 0% for the pre-mixed condition, and η = 100% for the completely mixed condition.

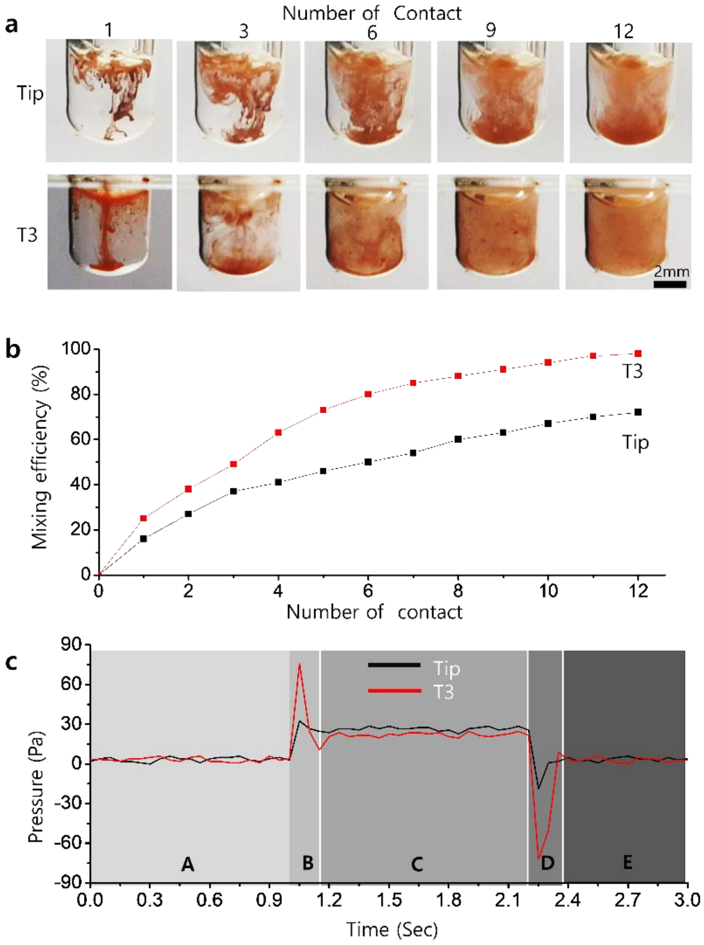

Figure S7. Mixing efficiency. (**a**) Photographs that show the mixing process. (**b**) Mixing efficiency in the bottom chamber. As the number of contacts between the bottom chamber and T3/tip is increased, the mixing efficiency increases. (**c**) Pressure profiles of the bottom chamber during one cycle of agitation.

Off-chip Incubation

To perform an off-chip assay, H1N1 virus suspension of a 70 µL volume at a concentration of 3.2×10^{-1} HAU was added to an antibody–MB solution with a 70 µL volume (Figure S8). The solution was then vortexed (Micromixer MX2, FINEPCR) under different times of 0.5, 2 and 3 h. Next, the solution was washed three times with PBS. Afterwards, an antibody–QD^2 solution with a 70 µL volume was added and vortexed for 0.5, 2, and 3 h with subsequent three washing steps with PBS. Finally, the immune complexes were moved to a fresh PBS and were measured.

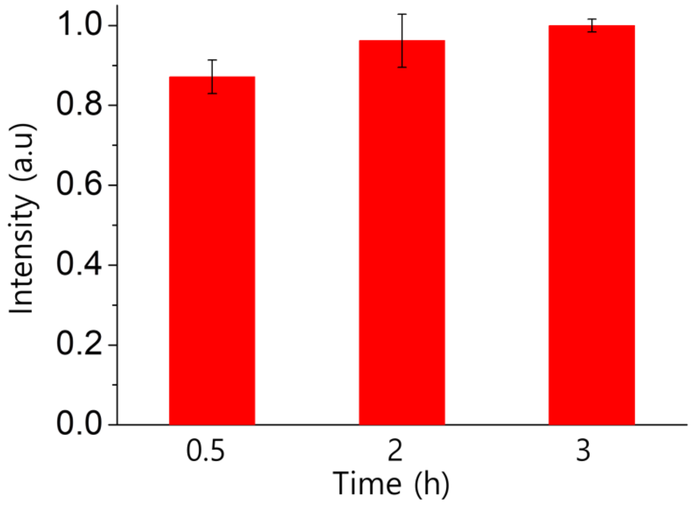

Figure S8. Change in the fluorescent intensity in the detection by off-chip incubation. The incubation time corresponds to the times in Steps 1 and 3 in each.

Biotin–Streptavidin Interaction

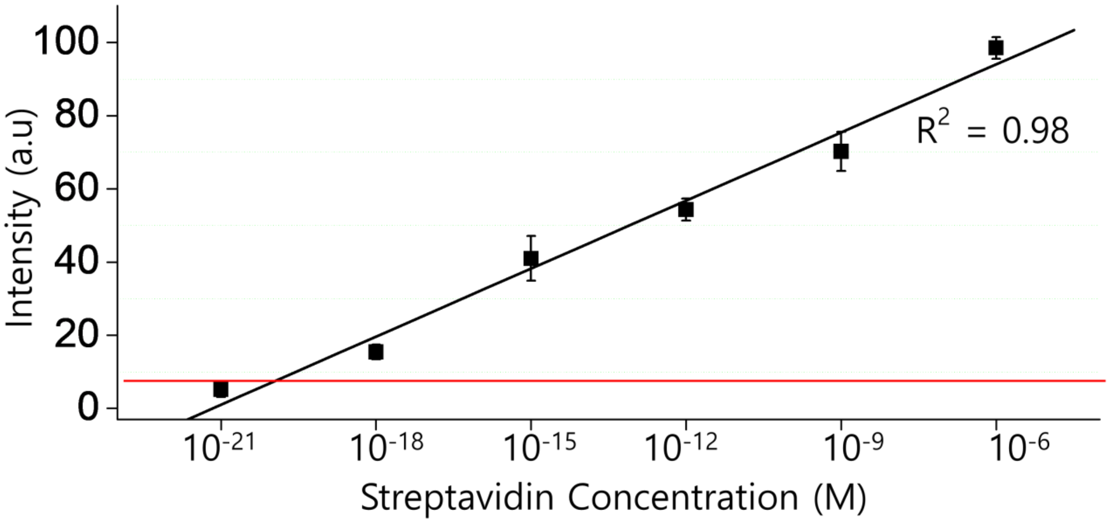

Figure S9. Fluorescence intensity for biotin–streptavidin interaction. The QD^2-based immunoassay was used as a model system to investigate the sensitivity. The red point shows the negative control (without streptavidin). The streptavidin LOD was found to be 0.39 aM. This result shows that the QD^2 and MB-based sandwich immunoassay with our device enables highly sensitive detection.

Table S1. Details of device component and its circuit.

Part	Component/ Material	Model	Company	Website (purchase store)
Circuit & optical detection	Microcontroller	ATmega328,	Arduino	https://eleparts.co.kr/goods/search?search_text=Arduino%20uno
	stepper motors (linear motion)	NEMA-23	HongYi Automation	https://es.aliexpress.com/item/4000079383138.html
	stepper motors (Rotational motion)	NEMA-17	Guangzhou Shenglong Motor	https://kr.element14.com/nanotec/st4118d3004-a/stepper-motor-3-3vdc-3a/dp/2507565?gclid=CjwKCAiAtouOBhA6EiwA2nLKH27ytNWGHMg19pcLWCtjpn1YXg7hWAkeJEUnRYsGN7KQYBf-2wanERoC4n0QAvD_BwE&mckv=_dc|pcrid|519456541117|pkw||pmt||slid||product|2507565|pgrid|119732683897|ptaid|pla-293946777986|&CMP=KNC-GKR-GEN-SMART-SHOPPING
	aspheric lens	83-677	Edmund Optics	https://www.edmundoptics.com/
	filter (620 nm)	FAS-Nano Amber Filter,	NIPPON Genetics	https://www.nippongenetics.eu/en/
	UV LED	VAOL-5EUV8T4,	Visual Communications Company	https://www.thorlabs.com/navigation.cfm?guide_id=2101&gclid=CjwKCAiAtouOBhA6EiwA2nLKH-LKDoi6qMrhuXEqB6OMwtxVj3TK8zP4F_5qxI4ZepQ_44hWnGY2MBoCESYQAvD_BwE
	Photomultiplier tube (PMT)	H10722,	Hamamatsu	https://www.hamamatsu.com/us/en/product/type/H10722-20/index.html
Layer and chamber component	Disposable top & bottom chambers & the tip	Resin: TAN V2 3D-printer:(Form 3)	Apply Labwork Formlabs	https://formlabs.com/asia/
	Permanent top and bottom layers	Resin: silver metallic PLA 3D-printer: Ultimaker3,	Ultimaker BV	https://ultimaker.com/
	Detection chamber	Transparent 200-μL tube	Applied Biosystems	https://www.thermofisher.com/order/catalog/product/4323032
	Permanent magnet	ND 3 (ϕ) × 15 (l) mm^2	Zion magnetic	http://item.gmarket.co.kr/Item?goodscode=192626120
Material & reagents	Magnetic bead	Polystyrene-based beads -(ϕ) 7.5 μm	Thermo fisher	www.thermofisher.com
	Quantum dot	CdSe@ZnS	Zeus	http://globalzeus.com/kor/product/product_list_ex.asp?cate0=435&cate1=614
	Washing buffer (PBS) and bovine serum albumin (BSA)	----------	Sigma Aldrich	https://www.sigmaaldrich.com/KR/ko?gclid=CjwKCAiAtouOBhA6EiwA2nLKH0Wwk3ZLF7muQ3LRW0QlVfNx7dPKOue8Xo_QIob53dWA-d0-f8kIrxoChyEQAvD_BwE

Table S2. Immunoassays using optical nanoprobes for virus detection.

Materials	Merit	LODs	Time	Ref
Gold immune chromatographic assays	Convenient and fast	500 pM–10 nM	Minutes	(2017). Lateral flow assay based on paper–hydrogel hybrid material for sensitive point-of-care detection of Dengue virus. Adv. Healthc. Mater. 6, 1600920. 40.
Magnetic particle Based Chemiluminescence (CL) immunoassays	Highly sensitive	50 fM–10 pM	Hours	(2006). A chemiluminescent, magnetic particle-based immunoassay for the detection of hepatitis C virus core antigen in human serum or plasma. J. Med. Virol. 78, 1436–1440.
Organic fluorescent molecules-based assays	Relatively Convenient	25 pM–1 nM	Minutes	(2015). Single-layer transition metal dichalcogenide nanosheet-based nanosensors for rapid, sensitive, and multiplexed detection of DNA. Adv. Mater. 27, 935–939.
UCNPs-based biosensors	Highly sensitive	60 fM–10 pM	Minutes to hours	(2016) Ultrasensitive detection of Ebola virus oligonucleotide based on upconversion nanoprobe/nanoporous membrane system. ACS Nano 10, 598–605.
QDs-based biosensors	Cost effective	1–200 nM	Minutes to hours	(2009). Positively charged compact quantum Dot-DNA complexes for detection of nucleic acids. ChemPhysChem 10, 806–811. (2016) Ultrasensitive detection of Ebola virus oligonucleotide based on upconversion nanoprobe/nanoporous membrane system. ACS Nano 10, 598–605.
QD2-magnetic bead based assay	Highly sensitive	< 1 pM	1 hour	(2020) Journal of Industrial and Engineering Chemistry, 90, 319-326
Automated QD2-magnetic bead based assay	Highly sensitive Relatively fast	< 1 pM	33 minutes	This study
